# Improved Finite Element Model Updating of a Highway Viaduct Using Acceleration and Strain Data

**DOI:** 10.3390/s24092788

**Published:** 2024-04-27

**Authors:** Doron Hekič, Diogo Ribeiro, Andrej Anžlin, Aleš Žnidarič, Peter Češarek

**Affiliations:** 1 Faculty of Civil and Geodetic Engineering, University of Ljubljana, Jamova cesta 2, 1000 Ljubljana, Slovenia; peter.cesarek@fgg.uni-lj.si; 2 Department of Structures, Slovenian National Building and Civil Engineering Institute, Dimičeva ulica 12, 1000 Ljubljana, Slovenia; andrej.anzlin@zag.si (A.A.); ales.znidaric@zag.si (A.Ž.); 3 CONSTRUCT-LESE, School of Engineering, Polytechnic of Porto, 4249-015 Porto, Portugal; drr@isep.ipp.pt

**Keywords:** finite element model updating (FEMU), optimisation, calibration, monitoring, concrete highway viaduct, structural health monitoring (SHM), error-domain model falsification (EDMF)

## Abstract

Most finite element model updating (FEMU) studies on bridges are acceleration-based due to their lower cost and ease of use compared to strain- or displacement-based methods, which entail costly experiments and traffic disruptions. This leads to a scarcity of comprehensive studies incorporating strain measurements. This study employed the strain- and acceleration-based FEMU analyses performed on a more than 50-year-old multi-span concrete highway viaduct. Mid-span strains under heavy vehicles were considered for the strain-based FEMU, and frequencies and mode shapes for the acceleration-based FEMU. The analyses were performed separately for up to three variables, representing Young’s modulus adjustment factors for different groups of structural elements. FEMU studies considered residual minimisation and the error-domain model falsification (EDMF) methodology. The residual minimisation utilised four different single-objective optimisations focusing on strains, frequencies, and mode shapes. Strain- and frequency-based FEMU analyses resulted in an approximately 20% increase in the overall superstructure’s design stiffness. This study shows the benefits of the intuitive EDMF over residual minimisation for FEMU, where information gained from the strain data, in addition to the acceleration data, manifests more sensible updated variables. EDMF finally resulted in a 25–50% overestimated design stiffness of internal main girders.

## 1. Introduction

The bridge management sector is facing many challenges strongly linked to climate change, which, in recent years, has accelerated the rate of material and structural degradation. For example, increased temperatures strengthen the corrosion rates [1] and amplify other risks [2], posing a significant threat to bridges’ safety and durability. Despite the uncertainties associated with the magnitude of the changes [3], it is accepted that they negatively affect infrastructure [4], which is subjected to longer and warmer dry spells and more frequent and severe flooding events, leading to economic losses [5].

The increased traffic capacity demands add to the challenges. ITF Transport Outlook states that tonne-kilometres of freight traffic worldwide will nearly double between 2019 and 2050 [6]. Furthermore, under the current ambition scenario, the share of road modes will increase from 22% to 27% in 2050. Traffic count data near the case study viaduct, designated in the following as the Ravbarkomanda viaduct, show that 3.6 million vehicles over 3.5 tonnes crossed the viaduct in 2022 [7,8], nearly a three-time increase since 2002 when 1.3 million vehicles had been recorded.

At times of increasing loads, the infrastructure is ageing. The average age of European and other developed countries’ bridges exceeds 50 years, as indicated in [9], affecting their condition. Many bridges before 1970 were designed for a service life of 50 years and are thus approaching the end of their design life [10]. Moreover, once considered long-lasting, reinforced concrete structures have not met these expectations, particularly those built in the 1970s [11,12,13]. A 2019 review [14] reported that 12% of highway bridges in Germany were in a very poor, insufficient, or inadequate condition, a figure that a 2022 report [15] has updated to nearly 13%.

Joint Research Centre (JRC) Science for Policy report [16] states that Europe’s ageing transport infrastructure needs effective and proactive maintenance to ensure its safe operation throughout its entire life cycle and ensure sufficient serviceability and safety. This can be achieved with adequate investments in inspections and structural health monitoring (SHM) systems and by prioritising interventions for critical structures with sustainable retrofitting solutions. Applying such an approach requires further research, particularly in benchmarking different SHM concepts. This is vital for standardisation and making informed decisions about the most suitable solutions for various applications, as initiated in the recent EU project IM-SAFE [17]. In the wake of significant events like the Morandi bridge collapse in Genoa, Italy [18], it has embarked on one of the most extensive SHM campaigns to date [19]. Such projects facilitate real-time monitoring that supplies critical data to assure safety and structural integrity.

Ageing infrastructure and increasing loads underline the necessity for preventive maintenance and inspection, visually and through SHM. Within SHM, various finite element model updating (FEMU) strategies are employed based on static and/or dynamic responses. Ereiz et al. [20] provide general guidelines about using SHM data to perform FEMU accurately. The process of FEMU is described step by step, namely (i) the selection of updating parameters (design variables); (ii) the definition of the model updating problem; and (iii) the solution of the model updating problem using different methods, particularly sensitivity-based, maximum likelihood, nonprobabilistic, probabilistic, response surface, meta heuristic, and regularisation methods.

Traditionally, FEMU and the damage detection of bridges are based on modal parameters (i.e., acceleration-based methods), using natural frequencies and mode shapes [21,22,23,24,25]. However, modal parameters may be limited because structures under traffic loads experience much larger amplitude responses than those under ambient ones. Also, bridges often experience light/moderate nonlinear incursions, particularly at the bearing devices [26], the track–deck interface [27], and the pavement–deck interface [28], among others. To overcome these limitations, several authors included in the FEMU problem static responses (displacements and strains) [29], dynamic responses (accelerations, displacements, and strains) [30,31], or a combination of these, mainly under traffic loads. Most updated models are used for the continuous condition assessments of bridges, particularly damage identification.

Comparative studies considering data from different sensor types (accelerometers, displacement sensors, strain sensors, etc.) or other types of tests (static and dynamic) are sparse. This paper contributes to a deeper perception of the differences between acceleration- and strain-based FEMU strategies. Understanding these differences is vital as the already established technologies are re-emerging, such as using bridge weigh-in-motion (B-WIM) for SHM, as proposed in [32]. Moreover, this paper contributes to understanding how the gradual increase in the number of variables affects the FEMU results. Lastly, the error-domain model falsification (EDMF) methodology, which was adopted for FEMU, in addition to the residual minimisation methodology, proved crucial, as it allowed for gaining critical insights into the updated values of variables. Despite its success, EDMF is still underused for FEMU. Hence, this study supports using EDMF for FEMU in civil structures, such as highway bridges.

## 2. Materials and Methods

### 2.1. Description of the Viaduct

**Figure 1:**
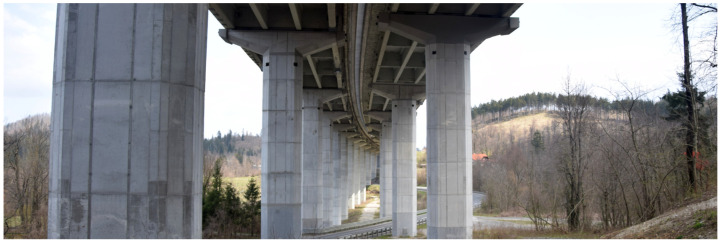
A view under the case study viaduct.

The case study, the Ravbarkomanda highway viaduct, is located in the southwestern region of Slovenia. It is over 50 years old, 560 m long, and comprises a 16-span precast I girder-type superstructure (Figure 1).

**Figure 2:**
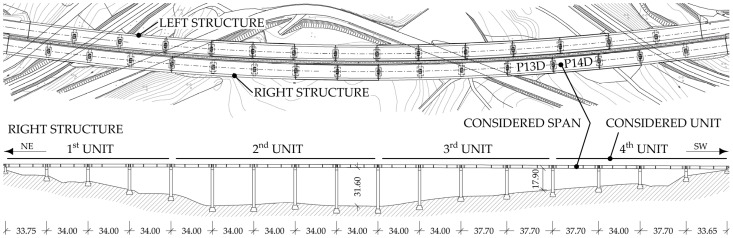
Plan view of both Ravbarkomanda viaduct structures and side view of the right structure with a notation of the P14D span and 4th unit, considered in this FEMU study (adapted from [32]).

As shown in Figure 1 and Figure 2, the viaduct consists of two parallel independent structures, the left carrying traffic northeast and the right one in the opposite direction. Each structure is divided into units bounded by expansion joints on both sides. Each of the two structures has four units. Precast I girders are discontinued above the piers, i.e., each girder bridges only one span, and the slab is continuous over the piers, except at expansion joint locations [33]. A detailed description of the viaduct and the established long-term monitoring that includes a B-WIM system can be found in [32].

This paper focuses on the fourth unit and the P14D span of the right structure, denoted as the *viaduct* throughout the paper. The paper follows the concept from a separate study [32], where strain-based FEMU was performed on the P14D span. This span was selected due to its extensive array of installed strain-gauge sensors, the largest of any span. A B-WIM system is also installed in this span to collect axle loads and spacings of all crossing vehicles.

### 2.2. Measurements of Strains under Passages of Calibration Vehicles

Strains were measured under crossings of three different calibration vehicles, designated V1 (two-axle rigid truck), V2, and V3. Both V2 and V3 were two-axle tractors with a three-axle semi-trailer. The calibration vehicles’ passages were performed primarily to calibrate the B-WIM system installed in the P14D span. Their axle loads and gross vehicle weights (GVWs) were preweighted statically, and their axle spacings were measured manually. The results are shown in Table 1.

**Figure 3:**
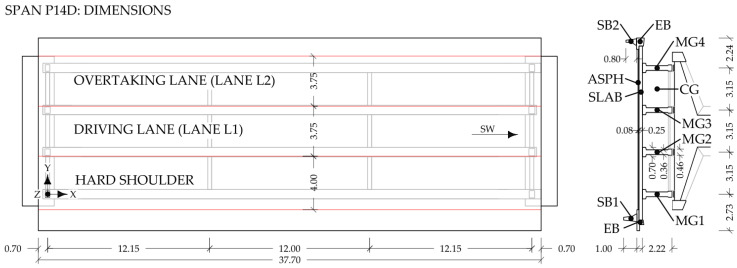
Plan view and cross-section of the P14D span with dimensions and notations of the structural and nonstructural elements: MG1–MG4 denote main girders, CG refers to cross-girders, SB1 and SB2 refer to safety barriers, EB refers to edge beam and SLAB denotes slab (adapted from [32]).

Vehicles V1, V2, and V3 crossed the structure in the driving lane (Figure 3) 16, 17, and 18 times, respectively. Their response was measured by strain gauges installed at the mid-span of the bottom flange of the P14D span’s main girders, labelled in Figure 3 as MG1, MG2, MG3, and MG4.

**Figure 4:**
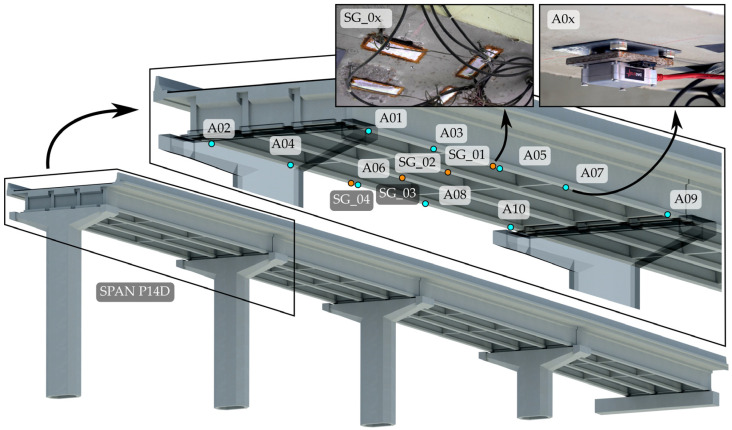
Render of a 4th unit with a detailed display of accelerometers and strain-gauge disposition in the P14D span.

Each girder had 2 or 3 nearby strain-gauge sensors installed near the mid-span. The manufacturer’s instructions were strictly followed in all installation stages: (concrete) surface preparation, glueing, protection, and connection of sensors. Two different types of strain gauges were used: TML PL-60-11-1LJC-F (120 Ω, half-Wheatstone type bridge, 60 mm gauge length; Tokyo Measuring Instruments Laboratory Co., Ltd., Tokyo, Japan) and Vishay C2A-06-20CLW-350 (350 Ω, half-Wheatstone type bridge, 50.8 mm gauge length; Vishay Intertechnology, Inc., Malvern, PA, USA). Signals from the girders were averaged to obtain more reliable strain responses per girder by reducing the errors due to possible uncertainties in location and faulty behaviour of the individual strain gauges. More is described in detail in Section 2.6.2 and in [32]. It is sufficient to assume that sensor SG_01 corresponds to the (average) measurements at the mid-span of girder MG1 and analogously applies to sensors SG_02, SG_03, and SG_04. Locations of sensors are shown in Figure 4, indicating that more strain-gauge sensors were installed at the same girder. Accelerometers are also shown in the figure, which is described in Section 2.3.

For the strain-based FEMU, described in Section 2.6.2, it was necessary to postprocess the strain measurements. The strain-based FEMU compared the measured strains to the FE-modelled ones under the calibration vehicles. Only the maximum values of the modelled and measured responses were compared, not the full-length signals. A separate study was performed to determine the position of all three vehicles that gave the greatest response at the strain-gauge sensor locations. Once determined, vehicles in the FE model were positioned in this location at every FEMU analysis. Such response under linear static analysis does not contain the dynamic component, and to compare it with the measured response, the latter should also be free of dynamics. The measured signals were, therefore, postprocessed with a 2 Hz low-pass filter to eliminate the dynamic component of the signal, thus obtaining the ‘pseudo-static’ response. A value of 2 Hz was selected based on a two-pass calculation of dynamic amplification factor (DAF) [34]. Table 2 shows the number of signals, mean, standard deviation, and coefficient of variation values for the maximum measured values in strain-gauge sensors.

### 2.3. Ambient and Traffic-Induced Vibration Tests

**Figure 5:**
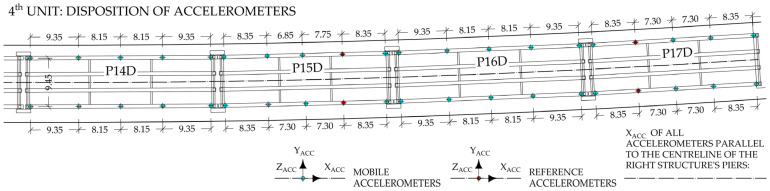
Plan view of the 4th unit with the disposition of the accelerometers during ambient and traffic-induced vibration tests; only Y_ACC_ and Z_ACC_ signals were used.

The long-term monitoring system installed on the viaduct does not include accelerometers on the superstructure. To perform the acceleration-based FEMU, additional short-term acceleration measurements were taken on the 4th unit. They were performed at 10 locations on the external main girders (MG1 and MG4) of the P14D span and on 30 more locations in the adjacent spans, namely P15D, P16D, and P17D (10 per span). Figure 5 introduces the measurement setup as a plan view of this unit, highlighting the placement of mobile and reference accelerometers. Measurements were performed in four setups; mobile sensors were moved between setups, and reference sensors remained in the same position during all setups.

Measurements were taken under a partial traffic closure; the hard shoulder was closed for traffic, and the driving lane (lane L1) was closed for traffic most of the time. During the measurements, the bridge experienced no congestion. However, trucks weighing over 3.5 tons were present, with an average frequency of one truck every 30 s.

For each setup, twelve Dewesoft type IOLITEi 3xMEMS-ACC triaxial MEMS accelerometers (Dewesoft, Trbovlje, Slovenia) [35] were used for approximately 30 min at a 1000 Hz sampling frequency. Accelerometers were attached on the lower side of the bottom flange of the main girders (Figure 4) via magnets and a steel plate glued to the concrete surface. DewesoftX 2023.5 data acquisition software [36] was used for data recording. Data were imported into the ARTeMIS Modal Pro 7.2 software [37] to estimate the modal parameters. Only measurements in the Y and Z directions, according to Figure 5, were used. Basic signal processing was performed before estimation, such as linear detrending and decimation to a new frequency range of [0–100 Hz]. The operational modal analysis (OMA) frequency domain decomposition (FDD) technique was used to extract the natural frequencies and mode shapes, where the spectra resolution was set to 1024 Hz, with a 66% overlap, representing a frequency resolution of 0.098 Hz.

**Figure 6:**
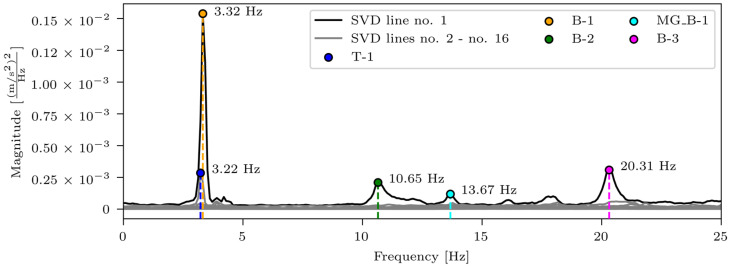
Singular values of spectral densities for the 1st test setup, with blue, orange, green, cyan, and magenta markers denoting 1st torsional mode (T-1), 1st bending mode (B-1), 2nd bending mode (B-2), 1st main girder local bending mode around the weak axis (MG_B-1), and 3rd bending mode (B-3).

The results of the first test setup, with eight mobile accelerometers installed in the P14D span and four reference accelerometers in the P15D and P17D spans, are shown in Figure 6. The figure presents singular values of spectral densities. It is annotated with different coloured markers for the identified modes: 1st torsional mode (T-1), 1st and 2nd bending modes (B-1 and B-2), 1st main girder local bending mode (MG_B-1), and 3rd bending mode. All modes except T-1 appear on the first (highest) SVD line.

Table 3 provides a comprehensive look at the identified natural frequencies and corresponding mode shapes from the experimental campaign. Mode shapes are shown in general and close-up views of the P14D span. Although five modes were identified, only four were considered for the acceleration-based FEMU. As shown in Figure 6, all modes are well separated, except the T-1 and B-1 modes, which are closely spaced. The T-1 mode, which appears on the second SVD line, and as such, is not the best estimate, according to [38], was omitted from the acceleration-based FEMU.

**Figure 7:**
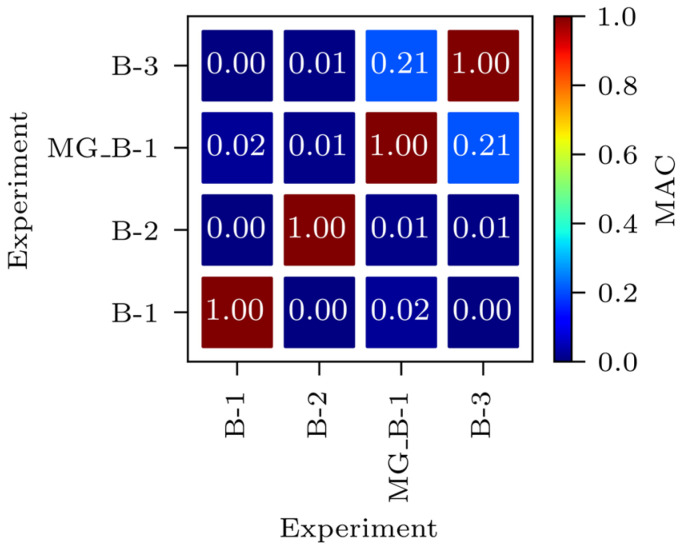
Auto-MAC matrix for the experimental mode shapes.

Figure 7 presents the auto-modal assurance criterion (Auto-MAC) matrix for the experimental mode shapes. MAC provides a measure of consistency (degree of linearity) between the considered mode shapes [39], for example, the modelled mode shapes with the measured ones. Auto-MAC is a version of the MAC used to compare mode shapes with themselves [40], in this case, experimental mode shapes.

It can be seen from Figure 7 that the experimental mode shapes of most nondiagonal values are close to 0, showing a low level of consistency (linearity), except for the MG_B-1 and B-3 modes, where the auto-MAC value is 0.21. The similarity of those two mode shapes can also be seen in Table 3.

### 2.4. Finite Element (FE) Model

**Figure 8:**
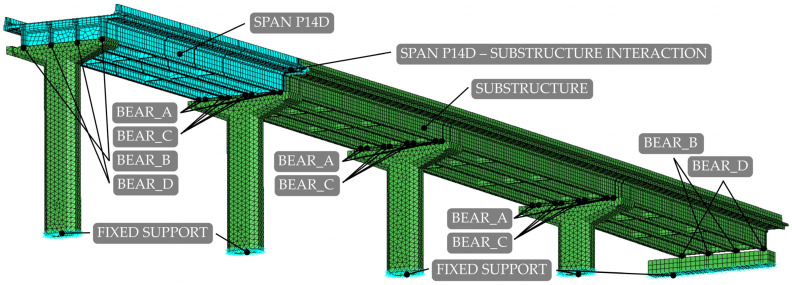
Initial finite element (FE) model M1_SUBSTR_INIT of the 4th unit.

The finite element (FE) model for the analysis of the 4th viaduct unit was developed in finite element analysis (FEA) software Abaqus 2019 [41] in two stages. First, the initial model (in the following designated as M1_FULL_INIT) was created, on which preliminary studies were performed. In the second stage, a model with reduced degrees of freedom (DOFs) was created (in the following designated as M1_SUBSTR_INIT), focusing on the P14D span, as shown in Figure 8. Besides the notations of the P14D span, substructure, supports, and location of the interaction between the P14D span and substructure, Figure 8 also shows the location and notations of the structural bearings, described in Section 2.4.2.

The main features of the initial model M1_FULL_INIT and its assumptions to form a model with a reduced number of DOFs M1_SUBSTR_INIT are outlined in Section 2.4.1, Section 2.4.2, Section 2.4.3 and Section 2.4.4.

#### 2.4.1. Geometry and Materials

The FE model followed the geometry from original design documentation [33,42], with minor simplifications of the edge beam. All elements were modelled with 3D solids and isotropic elastic material whose properties were taken from original design documentation [33,42] (Table 4).

#### 2.4.2. Interactions

The viaduct superstructure elements were assembled in one part, including the main girders, cross-girders, edge beams, slab, asphalt, and safety barriers. Consequently, their full interaction was assumed, and the safety barriers were treated as structural elements, fully contributing to the overall stiffness of the superstructure. A complex anchorage model to the viaduct deck would be required to model their contribution to the superstructure’s stiffness accurately, or reduction factors for their stiffness would need to be included in the FEMU process. The former would increase the computing time of the FEMU process, and the latter approach can yield a wide range of results, potentially complicating the overall outcomes of the FEMU process, as already discussed in [32]. Piers are connected to the superstructure with elastomeric bearings, modelled as wires (spring-dashpot assemblies) connecting reference points on the pier–girder contact surfaces. “Cartesian + Rotation” connector sections were assigned to these wires (assemblies), and their stiffness properties were obtained from [33]. Values of translational, vertical, and rotational stiffness for all four type of bearings were [3.10 × 10^3^ kN/m, 1.08 × 10^6^ kN/m, 3.09 × 10^3^ kNm] (BEAR_A); [2.43 × 10^3^ kN/m, 8.43 × 10^5^ kN/m, 2.32 × 10^3^ kNm] (BEAR_B); [3.72 × 10^3^ kN/m, 1.56 × 10^6^ kN/m, 7.32 × 10^3^ kNm] (BEAR_C); and [2.92 × 10^3^ kN/m, 1.22 × 10^6^ kN/m, 5.49 × 10^3^ kNm] (BEAR_D). Positions of the elastomeric bearings are shown in Figure 8.

#### 2.4.3. Boundary Conditions and Interaction with Adjacent Unit

The foundation of the piers is represented by fixing all translational degrees of freedom for the nodes on the bottom surface of the piers, as shown in Figure 8. The 4th unit interacts with the adjacent 3rd unit (Figure 2) only via a finger-type expansion joint. The adjacent span P13D, part of the 3rd unit, additionally restricts the movement of the shared pier that supports spans P13D and P14D. Therefore, in the M1_FULL_INIT model, the influence of the 3rd unit was modelled using connectors that link the locations of elastomeric bearings on the top of the pier with the ground. The stiffness properties of these connector sections were the same as the properties of the bearings they represented, except for translational stiffness in the X-direction, where the sum of the stiffness values in the X-direction of all bearings in 1st, 2nd, and 3rd unit was assumed.

#### 2.4.4. FE Mesh

Main girders, cross-girders, edge beams, slab, asphalt layer, and safety barriers were meshed using hexahedral 20-node quadratic (C3D20R) elements with a maximum global size of 0.50 m. Piers were discretised with 10-node quadratic tetrahedral elements (C3D10) with a maximum global size of 0.50 m. The maximum global element sizes were determined through a mesh convergence study. The global element sizes were gradually reduced, and the resulting natural frequencies and MAC values from different models were compared. The comparison was made for experimentally identified natural frequencies and corresponding mode shapes. Table 5 shows natural frequencies for FE models with 0.50 m and 0.25 m global element sizes.

**Figure 9:**
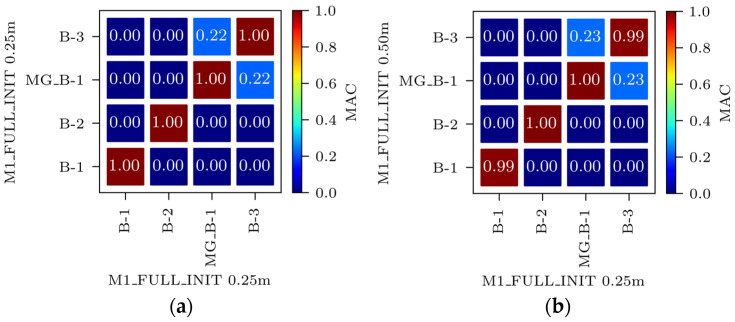
Mesh convergence study of the mode shapes for the M1_FULL_INIT FE model: Auto-MAC matrix for M1_FULL_INIT FE model with 0.25 m global element size (**a**) and MAC matrix for M1_FULL_INIT FE models with 0.50 m vs. 0.25 m global element size (**b**).

Figure 9a shows the Auto-MAC matrix for the M1_FULL_INIT model with a 0.25 m global element size. Figure 9b displays a MAC matrix for the M1_FULL_INIT model with global element sizes of 0.50 m and 0.25 m. Due to the balance of accuracy and computational efficiency, a global element size of 0.50 m was used.

**Figure 10:**
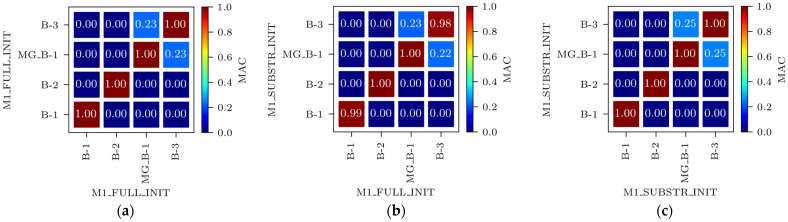
Auto-MAC matrix for M1_FULL_INIT FE model (**a**), MAC matrix for M1_SUBSTR_INIT vs. M1_FULL_INIT FE models (**b**), and Auto-MAC matrix for M1_SUBSTR_INIT FE model (**c**).

Even with larger finite elements, the M1_FULL_INIT model proved to be computationally intensive. To improve computational efficiency, a reduced-DOF model was created using the substructure modelling capabilities of Abaqus. In this context, “substructure” does not refer to the piers but to an entire structural component selected for separate analysis from the main structure. The P14D span was designated as the main structure, while the remaining parts of the 4th unit were modelled as a substructure (Figure 8). The substructure only contributes to the retained DOFs, including the supported nodes and nodes that interact with the main structure and provide stiffness of the substructure to the main structure during analysis. The reduced mass matrix and 90 retained modes of the substructure were computed to improve the accuracy of the main structure modal analysis (P14D span). The model with the substructure reduced the analysis time by 3.7 times compared to the M1_FULL_INIT FE model with 0.50 m global element size while maintaining the same level of result accuracy; natural frequencies of the M1_SUBSTR_INIT FE model (3.00 Hz, 9.83 Hz, 13.07 Hz, and 20.48 Hz) matched well with the M1_FULL_INIT FE model (3.00 Hz, 9.80 Hz, 12.78 Hz, and 20.37 Hz). Both models had a global element size of 0.50 m. Figure 10a shows the Auto-MAC matrix for the M1_FULL_INIT FE model, and Figure 10b displays a MAC matrix for M1_FULL_INIT and M1_SUBSTR_INIT FE models. Figure 10c shows the Auto-MAC matrix for the M1_SUBSTR_INIT FE model.

### 2.5. Comparison of the Initial FE Model M1_SUBSTR_INIT and Experiment

**Figure 11:**
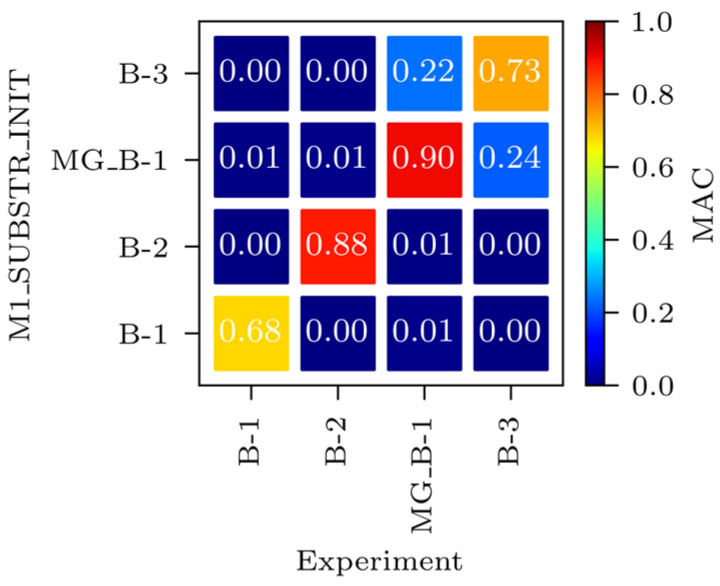
MAC matrix for M1_SUBSTR_INIT vs. experiment.

Table 6 compares natural frequencies and corresponding mode shapes of the M1_SUBSTR_INIT FE model and experimental values for all four modes considered within the acceleration-based FEMU: B-1, B-2, MG_B-1, and B-3. In addition, mode shapes were compared throughout the MAC matrix. From Figure 11, it is evident that the best match between the modelled and measured mode is for MG_B-1, with the MAC value amounting to 0.90. By contrast, the least similar are the B-1 mode shapes, with the MAC value of 0.68.

**Figure 12:**
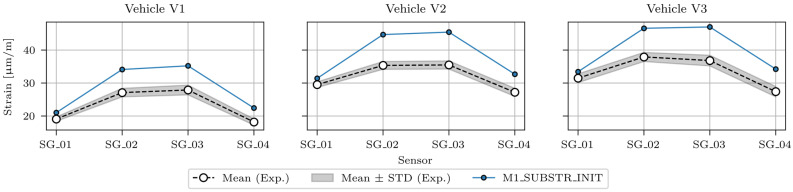
Maximum strains in the M1_SUBSTR_INIT FE model compared to the mean ± STD (standard deviation) values of maximum measured strains in sensors SG_01, SG_02, SG_03, and SG_04 under calibration vehicles V1, V2, and V3.

Comparison results for static analysis, where maximum strains under calibration vehicles were calculated and compared to the measured strains, are shown in Table 7, which compares the maximum modelled and measured strain values in sensors SG_01, SG_02, SG_03, and SG_04 (P14D span) under calibration vehicles V1, V2, and V3. In addition, for the measured strains, the STD (standard deviation) values are listed. Figure 12 graphically shows the values from Table 7.

Figure 12 shows how M1_SUBSTR_INIT overestimates responses in all sensors and for all vehicles. The overestimation is the smallest in SG_01 sensor (<10%), and the most significant one in SG_02 and SG_03 sensors (>20%, <30%).

### 2.6. Finite Element Model Updating (FEMU): Residual Minimisation

FEMU aims to reduce the difference between the modelled and measured response. Two approaches for large-scale structures are often used for FEMU, namely Residual minimisation and Bayesian interference, the first being considered in this study. Besides the residual minimisation approach, the less common EDMF methodology [43] was also performed in this study, which is described in Section 2.7.

A function that combines the measured and modelled responses is called an “index of discrepancy” or objective function. In this section, the objective functions used for the acceleration- and strain-based FEMU analyses are formulated, and the optimisation algorithm used for the automatic nonlinear single objective optimisation is presented.

#### 2.6.1. Objective Functions for Acceleration-Based FEMU

Three objective functions, namely $J_{f}$, $J_{MAC}$, and $J_{f,MAC}$, were considered for acceleration-based FEMU. The $J_{f}$ objective function measures the similarity of the modelled and measured natural frequencies. It is defined as follows:

(1) $$J_{f}=\sum_{i=1}^{4}{\left(\frac{f_{i,num}-f_{i,exp}}{f_{i,exp}}\right)}^{2},$$

where $f_{i,num}$ and $f_{i,exp}$ are the *i*th matching mode pair of the natural frequencies from the FE model experiment, respectively. According to the [44], this is the “normalised” $J_{2}$ type objective function. 

The $J_{MAC}$ objective function measures the similarity of the modelled and measured mode shapes. It is defined, similarly as in [22] or [45], as follows:

(2) $$J_{MAC}=\sum_{i=1}^{4}{\left(1-{MAC}_{i}\right)}^{2},$$

where ${MAC}_{i}$ compares the *i*th mode shape of the FE model with the *i*th reference experimental mode shape.

The $J_{f,MAC}$ objective function combines the $J_{f}$ and $J_{MAC}$ objective functions, similar to [22]. Since $J_{f}$ and $J_{MAC}$ are of different orders of magnitude, $w_{f}$ and $w_{MAC}$ weights were considered to ensure that contribution of both to the determination of $J_{f,MAC}$ would be comparable:

(3) $$J_{f,MAC}={w_{f}\cdot J}_{f}+{w_{MAC}\cdot J}_{MAC}.$$

The value of $w_{f}$ and $w_{MAC}$ were set to 11.3 and 1.0, respectively. The value of 11.3 represents the ratio of $J_{MAC}$ and $J_{f}$, calculated for the M1_SUBSTR_INIT FE model.

#### 2.6.2. Objective Function for Strain-Based FEMU

The $J_{\varepsilon }$ objective function is defined to measure the similarity of maximum modelled and measured strains at the mid-span of the P14D span when loaded by calibration vehicles V1, V2, and V3. It is defined as the sum of squared relative differences with standard deviation as a normalisation term. According to [44], this is the $J_{4}$-type objective function, with a minor modification, considering average responses in SG_01, SG_02, SG_03, and SG_04 sensors, as described in Section 2.2. The objective function $J_{\varepsilon }$ is defined as follows:

(4) $$J_{\varepsilon }=\sum_{v=1}^{n_{v}}\sum_{g=1}^{n_{g}}\frac{{\left(z_{num,v,g}-z_{exp,v,g}\right)}^{2}}{{{STD}_{exp,v,g}}^{2}}$$

where $z_{num,v,g}$ and $z_{exp,v,g}$ are calculated as follows:

(5) $$z_{num,v,g}=\frac{1}{n_{g,s}}\sum_{s=1}^{n_{g,s}}\varepsilon_{num,v,g,s}\;\mathrm{and}$$

(6) $$z_{exp,v,g}=\frac{1}{n_{g,s}}\sum_{s=1}^{n_{g,s}}\left(\frac{1}{n_{v,p}}\sum_{p=1}^{n_{v,p}}\varepsilon_{exp,v,g,s,p}\right).$$

The $z_{exp,v,g}$ and ${STD}_{exp,v,g}$ values are the “experimental” mean and STD values from Table 7. Individual terms in equations are described as follows:

- g denotes the main girder index;
- $n_{g}$ denotes the number of main girders considered (four in this study);
- $n_{g,s}$ denotes the number of strain gauges considered in a given girder *g* (two or three in this study);
- $n_{v}$ denotes the number of calibration vehicles considered (three in this study);
- $n_{v,p}$ denotes the number of vehicle *v* passages;
- p denotes the passage index of the selected calibration vehicle;
- s denotes the strain-gauge sensor index on the selected main girder;
- ${STD}_{exp,v,g}$ denotes the standard deviation of measured strains for main girder *g* and vehicle *v*;
- v denotes the calibration vehicle index;
- $\varepsilon_{exp,v,g,s,p}$ denotes the maximum measured longitudinal strain (section) in the sth strain-gauge sensor on the gth main girder, caused by the vth calibration vehicle during pth passage;
- $\varepsilon_{num,v,g,s}$ denotes the FE model longitudinal strain, oriented parallel to the X (longitudinal) direction of the viaduct$,\;\varepsilon_{XX}$, in the selected node that corresponds to the sth strain-gauge sensor on the gth main girder, caused by the vth calibration vehicle positioned on the location that results in the maximum strain at sensors SG_0*g*.

#### 2.6.3. Optimisation Algorithm

In this study, the particle swarm optimisation (PSO) algorithm [46] was used to update the FE model automatically, which is one of the most commonly used algorithms in FEMU [20]. For the automatic FEMU, it is advantageous if the FEA software can interact with external programming environments such as MATLAB, Python, and Mathematica. This interaction involves preparing input files for analysis, submitting the FEA job, examining the FEA outcomes (output files), and generating new input files based on the decisions of the optimisation algorithm. In this research, the Abaqus 2019 FEA software was employed, along with Python 3.10, using the scipy.optimise.minimise [47] and pymoo [48] libraries. All parameters of the PSO algorithm were set to default (according to [48]) for all FEMU analyses, except for the population size, which was set to 100, and 20 generations were set as the stop criteria.

### 2.7. FEMU: Error-Domain Model Falsification (EDMF)

EDMF is a methodology for structural identification, introduced for bridge load testing in 2013 [43] and applied in 2019 [49] and recently in 2023 [50]. The falsification concept, as stated by [43], has been well known in science for centuries but was formalised only in the 1930s by Karl Popper, who stated that, in science, models cannot be fully validated by data. Instead, they can only be falsified. EDMF identifies plausible values of the FE model variables (parameters) based on experimental values from field measurements and prescribed uncertainty levels. A population of FE model instances is generated where each instance has a unique combination of variable values. Then, the FE model predictions (responses) are compared with the sensor data collected during the experiment. FE model instances where the difference between the modelled and measured responses exceeds thresholds defined based on uncertainty levels are falsified (falsified models), and the rest are designated as candidates. Updated ranges of variables are obtained by discarding variable values from falsified model instances.

As stated by [51], using thresholds for falsification enables EDMF to be robust to correlation assumptions between uncertainties; moreover, EDMF explicitly accounts for model bias based on engineering heuristics. Consequently, EDMF, when compared with traditional Bayesian model updating and residual minimisation, has been shown to provide more accurate identification and prediction when there is significant systematic uncertainty. EDMF has been gaining popularity in recent years, since only between 2015 and 2022, there were nine case studies on bridges, four on buildings, and two on geotechnical excavations reported worldwide [51].

EDMF for the considered case study was primarily utilised to verify the suspicious FEMU results from the residual minimisation, particularly the final values of variables that reached the lower and upper bound of the preset range and were not in accordance with the engineering expectation.

## 3. Results

### 3.1. Sensitivity Study

A deterministic sensitivity study was performed to understand the impact of the individual structural elements on the values of objective functions $J_{f}$ and $J_{MAC}$. For the $J_{\varepsilon }$ objective function, the sensitivity study results from the reference P14D-span-only study [32] are shown.

#### 3.1.1. Variables

A sensitivity study for $J_{f}$ and $J_{MAC}$ was performed on the M1_SUBSTR_INIT FE model such that the variable of the selected element was set to lower and upper values. In contrast, the properties of all other elements in the model were kept constant. Variables and their lower and upper values are defined in Table 8.

For structural elements, the lower and upper values are defined as 0.75 and 1.25 times the design Young’s elastic modulus values, which are shown in Table 4. For elastomeric bearings, the lower and upper values are defined as 0.75 and 1.25 times the design stiffness, as shown in Section 2.4.2. To assess the influence of density variations on the structural elements, they were simultaneously adjusted to two different levels for all elements, i.e., to 0.95 (lower value) and 1.05 (upper value) times their design values (Table 4).

#### 3.1.2. Acceleration-Based FEMU

**Figure 13:**
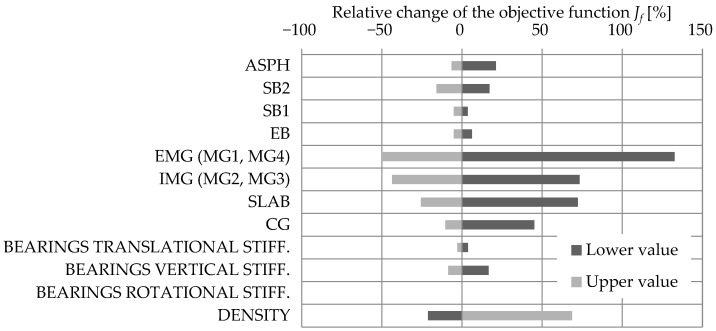
The sensitivity study results of the influence of structural elements Young’s modulus, bearing stiffness, and density on the relative change in the objective function $J_{f}$.

**Figure 14:**
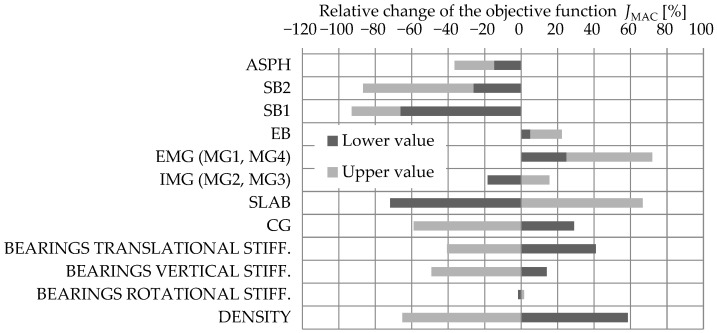
The sensitivity study results of the influence of structural elements Young’s modulus, bearing stiffness, and density on the relative change in the objective function $J_{MAC}$.

The results of the sensitivity study for the acceleration-based FEMU are shown separately for natural frequencies ($J_{f}$) and mode shapes ($J_{MAC}$). Figure 13 and Figure 14 show the sensitivity results where the FE model objective function, either taking a lower or upper value, is first summed up over all four modes considered and then compared to the summed-up objective function of the M1_SUBSTR_INIT FE model. The results are shown in % as a relative change compared to the M1_SUBSTR_INIT FE model. Such a representation gives a general insight into which variables contribute the most to the relative change in the objective function.

Figure 13 yields the conclusion that among all variables considered, the reduction in the objective function $J_{f}$ is the most sensitive to EMG, IMG, and SLAB elements’ increase in Young’s elastic modulus. Bearings do not have a significant impact. Figure 14, compared to Figure 13, is less concrete in suggesting which variables the objective function $J_{MAC}$ is most sensitive to. Reducing the objective function $J_{MAC}$ is mostly affected by SLAB and SB1 elements’ decrease in Young’s elastic modulus and by an increase in Young’s elastic modulus in CG and SB2. An increase in translational and vertical stiffness of elastomeric bearings, as well as a decrease in their density, importantly reduces the objective function $J_{MAC}$.

#### 3.1.3. Strain-Based FEMU

For the $J_{\varepsilon }$ objective function, the sensitivity study results are shown from the reference study, where only the P14D span was modelled. The interested reader is referred to [32] for a detailed description. The same structural elements and bearings were checked for sensitivity as for $J_{f}$ and $J_{MAC}$; only the density was omitted.

**Figure 15:**
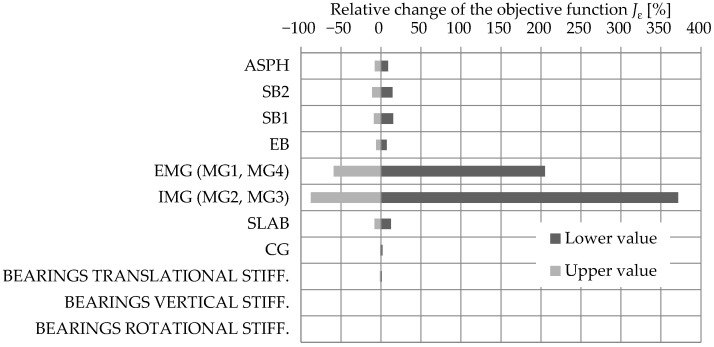
The strain-based sensitivity study results show the influence of structural elements Young’s modulus and bearing stiffness on the objective function value $J_{\varepsilon }$ (adapted from [32]).

As seen in Figure 15, the reduction in the objective function $J_{\varepsilon }$ is the most sensitive to EMG and IMG elements’ increase in Young’s elastic modulus. SB2, SB1, ASPH, and SLAB elements have comparable but much smaller influence.

#### 3.1.4. Variables Selected for FEMU

Based on the sensitivity study results, it was decided to update only Young’s modulus of structural elements and consider the constant design values of other properties. Furthermore, instead of updating Young’s modulus of individual structural elements, a grouping was performed such that Young’s modulus for all elements in the same group was updated for the same percentage/correction factor, in the following labelled as a “Young’s modulus adjustment factor”. Grouping was performed to observe the influence of several variables on the FEMU results. For the first FEMU studies, all structural elements were grouped. Thus, only one variable ($\alpha_{ALL}$) was updated. Later, the structural elements were regrouped into the EMG+IMG (MG) group and the OTHER group, consisting of all other elements. Two variables, $\alpha_{MG}$ and $\alpha_{OTHER}$ were updated in that case. Finally, the EMG+IMG (MG) group was split into EMG and IMG groups. Thus, three variables were updated: $\alpha_{ALL}$, $\alpha_{EMG}$, and $\alpha_{IMG}$. The variables and ranges within which the updated variables can take values are described in Table 9. 

It is important to emphasise that the goal of FEMU, as stated by [43], is not to update the model parameters to improve the agreement between predicted and measured values. Instead, model-based system identification uses physics-based models to infer parameter values. As such, the variables selected for FEMU do not represent the actual properties of the structural elements, i.e., Young’s modulus (adjustment factor). Instead, they should be treated as a mixture of structural properties condensed in a single variable. This needs to be kept in mind, especially when interpreting the absolute values of updated variables.

### 3.2. Updated FE Model

#### 3.2.1. List of Analyses

Twelve FEMU residual minimisation methodology analyses were performed. Four of them considered one variable ($\alpha_{ALL}$), four considered two variables ($\alpha_{MG}$, $\alpha_{OTHER}$), and the last four involved three variables ($\alpha_{EMG}$, $\alpha_{IMG}$, and $\alpha_{OTHER}$). In each group, four FEMU analyses were performed: frequency-based, MAC-based, frequency-and-MAC-based, and strain-based. All acceleration-based analyses considered B-1, B-2, MG_B-1, and B-3 modes, and all strain-based analyses considered all three calibration vehicles V1, V2, and V3. Three FEMU analyses considered EDMF methodology, all for three variables. One was acceleration-based, one was strain-based, and the last one was acceleration-and-strain-based methods. A summary of all these analyses is presented in Table 10.

#### 3.2.2. FEMU Results: Residual Minimisation

**Figure 16:**
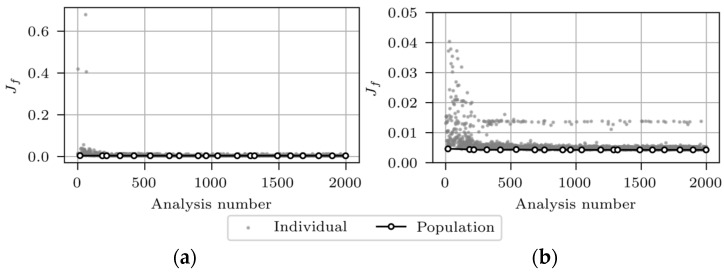
Evolution of the objective functions $J_{f}$ for analysis number 5, including all data (**a**) and zoomed-in view (**b**).

In this section, the results for all twelve FEMU analyses are presented, and prior to that, the evolution throughout the FEMU is shown for analysis number 5 (frequency-based analysis). Figure 16 illustrates the evolution of the objective function $J_{f}$ including all data (**a**) and using a zoomed-in view (**b**).

**Figure 17:**
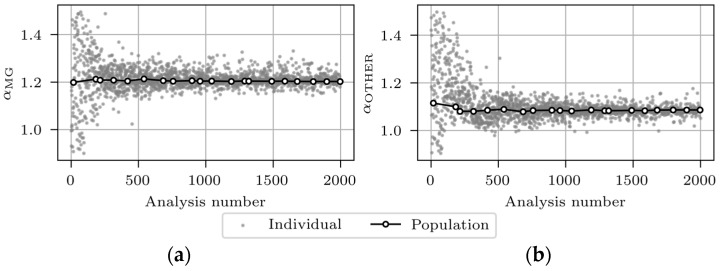
Evolution of $\alpha_{MG}$ (**a**) and $\alpha_{OTHER}$ (**b**) for FEMU analysis number 5.

The grey markers in Figure 16 represent the $J_{f}$ values of 2000 FE analyses, and the white markers denote the minimum $J_{f}$ values of each of the 20 data subsets (populations), each containing 100 results. The grey markers’ scatter decreases with the number of analyses. Some analyses, despite the high sequence number, even after the 1000th analysis, give a high $J_{f}$ value. This results from the incorrectly paired FE experimental modes, which could not be completely eliminated. Figure 17a,b present the evolution of $\alpha_{MG}$ and $\alpha_{OTHER}$, respectively.

The most significant variation in $\alpha_{MG}$ and $\alpha_{OTHER}$ occurs within the first 500 analyses and finally converges towards 1.20 and 1.09, respectively. Table 11 presents the FEMU results for all analyses of 12 separately updated FE “M1_SUBSTR_UPDATE_*i*” models, where *i* represents the analysis number. For each analysis, the final updated FE model was selected as the best individual from the final data subset (the last white marker, shown in Figure 16.

For single-variable analyses ($\alpha_{ALL}$), frequency-based, and frequency-and-MAC-based FEMU analyses give the same $\alpha_{ALL}$ value of 1.18, which matches the strain-based value of 1.21. MAC-based FEMU results differ considerably, with a value of 0.99.

For analyses with two variables ($\alpha_{MG}$ and $\alpha_{OTHER}$), a good match between the frequency- and strain-based FEMU is observed for $\alpha_{MG}$: The values are 1.20 and 1.17, respectively. MAC-based and frequency-and-MAC-based FEMU analyses for $\alpha_{MG}$ give comparable values of 0.96 and 0.99, which, however, are 20% lower than the frequency- and strain-based values. Contrary to $\alpha_{MG}$, FEMU gives very different frequency- (1.09) and strain-based $\alpha_{OTHER}$ (1.50) results. As all objective functions are more sensitive to $\alpha_{MG}$ than $\alpha_{OTHER}$, the better match for $\alpha_{MG}$ is reasonable.

For analyses with three variables ($\alpha_{EMG}$, $\alpha_{IMG}$, and $\alpha_{OTHER}$), a good match between the frequency- and strain-based FEMU is again observed for $\alpha_{EMG}$, namely 0.91 and 0.90, respectively. Additionally, MAC-based FEMU also gives a value of 0.90. Values of$\;\alpha_{IMG}$ are 1.50 for both frequency- and strain-based FEMU analyses, differing considerably from MAC-based (0.96) and frequency-and-MAC-based (0.93) FEMU.

It is evident from Table 11 for analyses with three variables that $\alpha_{EMG}$ reaches the lower bound (0.90), and $\alpha_{IMG}$ reaches the upper bound (1.50) of the preset range. One way to avoid variables reaching these bounds is to rerun FEMU analyses 9–12 with extended lower and upper bounds. The question of whether the results are reasonable in an engineering context arises when the range is too wide, i.e., there is a concern about whether a global minimum that does not reflect the physical properties of the considered problem is reached.

The following analysis pairs are expected to give comparable values of $\alpha_{OTHER}$: 5–9, 6–10, 7–11 and 8–12. These values are expected to be similar because the only difference between them is the split of the MG group into the EMG and IMG groups. Elements within the OTHER group remained unchanged. The previously mentioned analysis pairs do not give comparable values of $\alpha_{OTHER}$, due to the insensitivity coincidence of the objective functions to Young’s modulus of the elements in the OTHER group. Splitting the OTHER group into more subsets would increase the insensitivity even more. Therefore, three variables for the considered case study represent the sensible upper bound.

When comparing the FEMU results, it is important to stress the influence of temperature during the experiments. Strain and acceleration measurements were not taken simultaneously. The latter was obtained at a later stage, with the ambient temperature roughly 3 °C above the average 15 °C recorded during the strain measurements. According to [52], such a temperature difference would cause an insignificant 1% decrease in Young’s elastic modulus of concrete at higher temperatures.

Table 12 shows values of measured, initial model’s (M1_SUBSTR_INIT), and updated model’s (M1_SUBSTR_UPDATE_*i*) natural frequencies for frequency-based, MAC-based, and frequency-and-MAC-based FEMU analyses. As expected, the match between the modelled and measured frequencies is the best for the frequency-based FEMU (analyses 1, 5, and 9). MAC-based FEMU (analyses 2, 6, and 10) generally underestimates the first three frequencies by approximately 10%. Frequency-and-MAC-based FEMU analyses 3 and 11 give good matches for all four natural frequencies, comparable to frequency-based FEMU analyses 1 and 9, respectively. By contrast, analysis 7 gives a poor match for frequencies—in between the frequency-based analysis 5 and MAC-based analysis 6.

**Figure 18:**
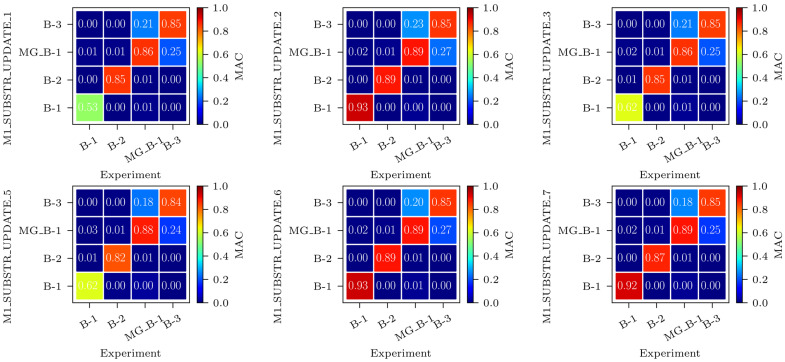

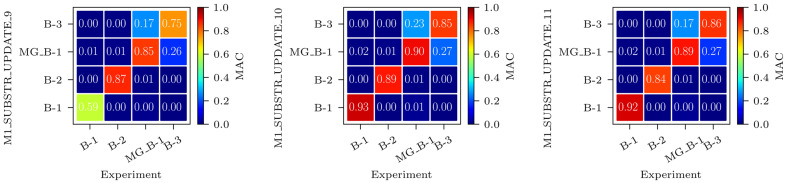
MAC matrices for frequency-based, MAC-based, and frequency-and-MAC-based FEMU analyses: M1_SUBSTR_UPDATE_*i* FE model vs. experiment.

Figure 18 shows the MAC matrices for frequency-based, MAC-based, and frequency-and-MAC-based FEMU analyses. Nine MAC matrices are shown, where mode shapes of the FE models M1_SUBSTR_UPDATE_*i* are compared to the experimental mode shapes. The modelled and experimental mode shapes match the best for MAC-based FEMU (analyses 2, 6, and 10) and the worst for frequency-based FEMU (analyses 1, 5, and 9). The most significant difference in the MAC values can be seen for the B-1 mode shape; while frequency-based FEMU analyses give MAC values between 0.53 and 0.62, MAC-based FEMU analyses give MAC values of 0.93. Frequency-and-MAC-based FEMU analyses 7 and 11 provide a good match for all mode shapes, comparable to MAC-based FEMU analyses 2 and 8, respectively. By contrast, analysis 3 results in a poor match.

**Figure 19:**
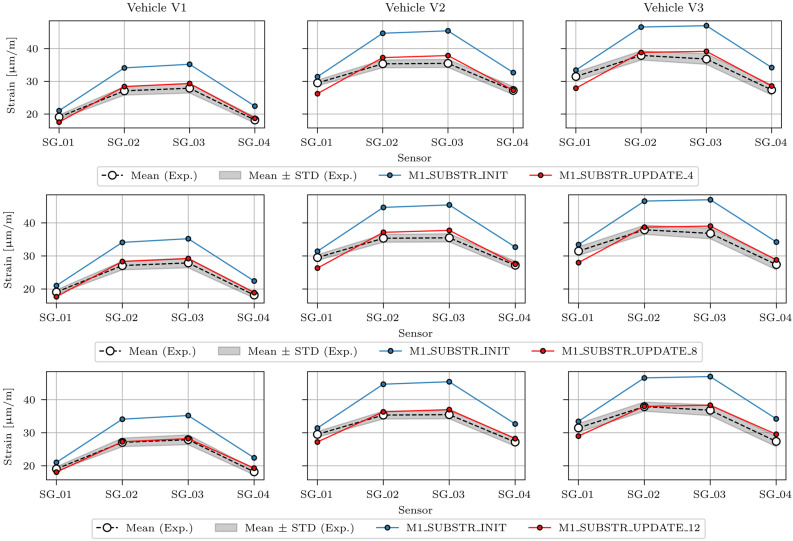
Maximum strains in the M1_SUBSTR_UPDATE_4, M1_SUBSTR_UPDATE_8, and M1_SUBSTR_UPDATE_12 FE models compared to the M1_SUBSTR_INIT model and mean ± STD (standard deviation) values of maximum measured strains in sensors SG_01, SG_02, SG_03, and SG_04 under calibration vehicles V1, V2, and V3.

Figure 19 shows the maximum strains for the strain-based FEMU (analysis numbers 4, 8, and 12), compared to the maximum strains of the M1_SUBSTR_INIT FE model and mean ± STD (standard deviation) values of the maximum measured strains in sensors SG_01, SG_02, SG_03, and SG_04 under the calibration vehicles V1, V2, and V3. The best match was achieved for FEMU analysis number 12, and the poorest match was recorded for FEMU analysis number 4.

#### 3.2.3. FEMU Results: EDMF

The EDMF methodology was adopted for FEMU considering the three variables, in addition to the residual minimisation analyses 9–12, to gain a more comprehensive insight into the problem. This allowed for more detailed insight into which variables and how they affect the FE model’s response. As stated by [43], model simplifications are always present when modelling full-scale civil structures, and the relationship between errors is usually unquantifiable. Model simplifications usually come, among others, from the omission of load-carrying elements (in this study, safety barriers) or improper distribution of loads (in this study, the position of calibration vehicles and the filtration of the dynamic strain signal).

EDMF results in this section are shown separately for acceleration-based, strain-based, and acceleration-and-strain-based FEMU analyses. Initially, 9464 FE models with a unique combination of variable values $\alpha_{EMG}$, $\alpha_{IMG}$, and $\alpha_{OTHER}$ were calculated for static analysis (strain-based FEMU) and modal analysis (acceleration-based FEMU). The range for the variables was intentionally set to be wider than for analyses 9–12. This was carried out to show the models with physically unacceptable variable values and how EDMF methodology can help avoid them. The set of variable values was the same for $\alpha_{EMG}$ and $\alpha_{IMG}$. Each can take 26 different values: the minimum value of 0.10 (lower bound) and the maximum value of 2.00 (upper bound). The range for $\alpha_{OTHER}$ was defined between the lower bound of 0.10 and the upper bound of 1.90 (14 values overall). As shown in Table 13, the intervals between values are not uniform. To optimise the number of FE analyses, the range for $\alpha_{OTHER}$ was, based on the sensitivity study results, sparser than for the $\alpha_{EMG}$ and $\alpha_{IMG}$. After the FE analyses were performed, falsification thresholds were defined.

For acceleration-based EDMF, falsification thresholds were defined for natural frequencies and MAC values of the mode shapes for all four modes. Threshold values were defined iteratively, initially allowing natural frequencies to deviate ±5% relative to the experimental values, and the absolute MAC values being at least 0.90, following the recommendation by [53] of a ‘good correlation’ between the FE model and experiment. For strain-based EDMF, the initial falsification thresholds were set to ±5% relative to the experimental values in sensors SG_01, SG_02, SG_03, and SG_04. Table 14 shows the final falsification threshold values, modified from the initial ones, to obtain enough candidates. Too narrow thresholds could lead to too few or even no candidates, and too loose ones could give too many.

Finally, the results of all FE models were tested for the fit within the falsification threshold bounds for the following factors:

- All frequencies and all MACs (acceleration-based EDMF, analysis number 13);
- All strains (strain-based EDMF, analysis number 14);
- All frequencies, all MACs, and all strains (acceleration-and-strain-based EDMF, analysis number 15).

**Figure 20:**
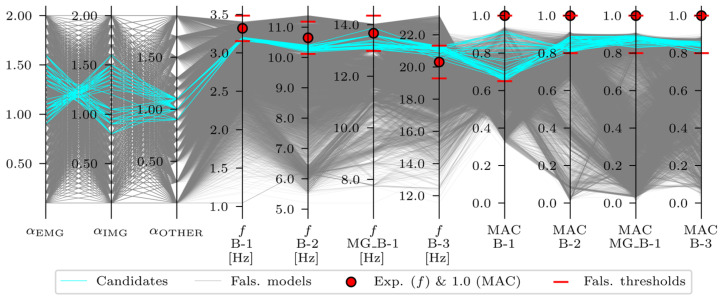
Acceleration-based EDMF results.

Only the FE models that fit all threshold bounds were designated as acceleration-based, strain-based, or acceleration-and-strain-based candidates and were included in the candidate model set, meaning their variable values are plausible. The last step was to critically overview the candidate model sets in terms of whether the results were meaningful in an engineering context. The acceleration-based EDMF results are shown in Figure 20.

**Figure 21:**
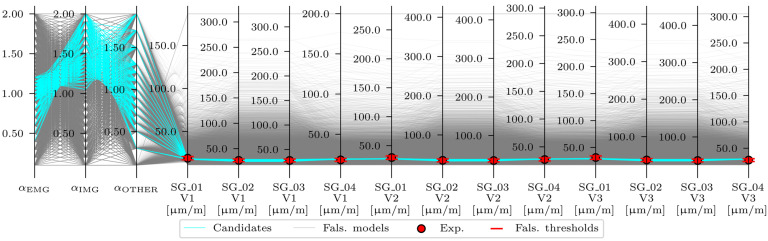
Strain-based EDMF results.

Overall, 35 candidates that fit into all threshold bounds for acceleration-based EDMF were identified (Table 15). However, not all of them were final, engineering-feasible candidates. Recalling the partially connected safety barriers, modelled as structural elements (described in Section 2.4.2 and [32]) and positioned close to the EMG elements, it was expected that this would manifest in the $\alpha_{EMG}$ lower than the $\alpha_{IMG}$. Therefore, only the candidate model sets with $\alpha_{EMG}<\alpha_{IMG}$ were expected to be the final candidates. The strain-based EDMF results are shown in Figure 21.

**Figure 22:**
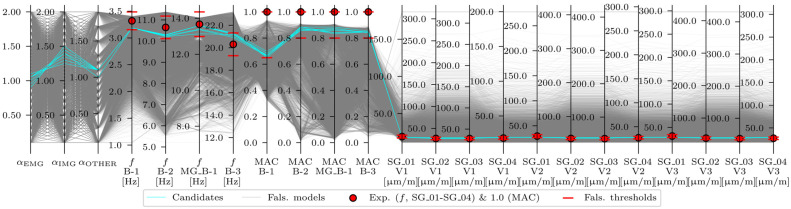
Acceleration-and-strain-based EDMF results.

Overall, 199 candidates were identified for the strain-based EDMF. Most (191) satisfied the $\alpha_{EMG}<\alpha_{IMG}$ criteria. Although the number of candidates was reduced from 9464 to 199 (≈2%), the range of the variables after strain-based EDMF, shown in Table 15, was still wide, especially for $\alpha_{OTHER}$. Figure 22 shows the acceleration-and-strain-based EDMF results.

**Figure 23:**
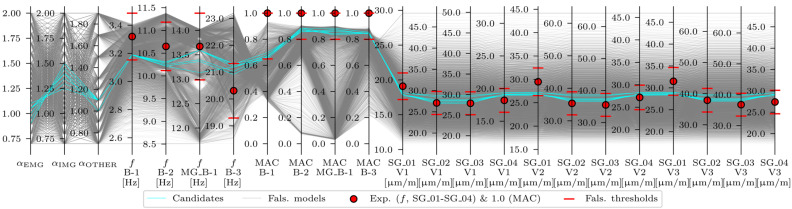
Acceleration-and-strain-based EDMF results, shown for a limited range of model sets with values of $\alpha_{EMG}$ *≥* 0.7, $\alpha_{IMG}$ *≥* 0.7, and $\alpha_{OTHER}$ *≥* 0.7.

Overall, seven candidates were identified for the acceleration-and-strain-based EDMF. All seven candidates satisfied the $\alpha_{EMG}<\alpha_{IMG}$ criteria and the updated range of the variables was narrowed significantly compared to the acceleration-based EDMF and strain-based EDMF, as shown in Table 15. Moreover, none of the variables reached the lower and upper bounds of the predefined range. As experimental values and falsified thresholds for strains are not clearly visible in Figure 22, a similar plot in Figure 23 shows a limited range of model sets with values of $\alpha_{EMG}$ *≥* 0.7, $\alpha_{IMG}$ *≥* 0.7, and $\alpha_{OTHER}$ *≥* 0.7.

With the provided falsification thresholds, both acceleration-based EDMF and strain-based EDMF significantly reduced the number of candidate model sets from an initial 9464 to 35 (0.4%) and 199 (2%), respectively. However, acceleration-based EDMF included engineering unacceptable candidate model sets with values of $\alpha_{EMG}$ greater than $\alpha_{IMG}$. This was also true for a very small proportion of candidate model sets given by strain-based EDMF. Only the candidate model sets, given by the acceleration-and-strain-based EDMF, contained engineering-acceptable values of $\alpha_{EMG},$ $\alpha_{IMG}$, and $\alpha_{OTHER}$. The deviation between ranges is mainly attributed to the safety barriers SB1 and SB2, which, although modelled as fully connected to the superstructure, are only partially connected.

Although the 3D finite elements allow for a high level of the FE model detailing, the systematic biases in the FE model remain present, resulting from the partially connected safety barriers and the unknown exact position of the calibration vehicles. EDMF methodology is computationally more demanding for the FEMU than the residual minimisation. Nevertheless, it was the key for the case study, as it allowed for combining acceleration- and strain-based FEMU studies and making an engineering decision about their updated values.

## 4. Conclusions

This paper presents the results of multiple FEMU studies of a highway viaduct that considered both strain responses under the traffic loading and accelerations from the traffic-induced and ambient vibration tests. The updated parameters from these two types of tests were compared. Furthermore, the residual minimisation FEMU approach was combined with the EDMF methodology. Despite being known to perform well in system identification, the latter is still underused in FEMU, compared to residual minimisation and Bayesian interference.

This study focused on updating structural parameters through Young’s elastic modulus of different groups of superstructure elements, e.g., all members, main, external main, or internal main girders. A dozen FEMU analyses were performed considering residual minimisation methodology. Four of them considered one variable ($\alpha_{ALL}$), four considered two variables ($\alpha_{MG}$, $\alpha_{OTHER}$), and the last four considered three variables ($\alpha_{EMG}$, $\alpha_{IMG}$, and $\alpha_{OTHER}$*).* Four separate FEMU analyses were performed for each number of variables: frequency-based, MAC-based, frequency-and-MAC-based, and strain-based. Acceleration-based analyses considered four modes (natural frequencies and mode shapes), while strain-based analyses considered the maximum strains measured under three calibration vehicles. Frequency- and strain-based FEMU studies for the single variable $\alpha_{ALL}$ yielded comparable values of 1.18 and 1.21. For analyses with two variables ($\alpha_{MG}$ and $\alpha_{OTHER}$), a good match between the frequency- and strain-based FEMU was observed for $\alpha_{MG}$: 1.20 and 1.17. For analyses with three variables, $\alpha_{EMG}$ reached the lower bound (0.90), and $\alpha_{IMG}$ reached the upper bound (1.50), in frequency- and strain-based FEMU analyses. Three additional FEMU analyses for three variables, applying the EDMF methodology, yielded engineeringly sensible results for the considered problem. The last EDMF analysis, which combined acceleration and strain data, proved to be crucial; initial ranges of variables were narrowed to [0.90, 1.10] for $\alpha_{EMG}$, [1.25, 1.50] for $\alpha_{IMG}$, and [1.00, 1.10] for $\alpha_{OTHER}$.

The results of this study show that frequency- and strain-based FEMU similarly overestimated the superstructure’s design bending stiffness by approximately 20%. When the main girders were separated from other elements, both methods again overestimated the design bending stiffness of the main girders by approximately 20%. When the main girders were additionally split into external and internal ones, the acceleration- and strain-based EDMF overestimated the internal main girders’ design bending stiffness by 25–50%. No significant overestimation was obtained for the external main girders, most likely due to the partially connected safety barriers.

The key advantages of the EDMF methodology over residual minimisation are highlighted in this study, particularly its intuitiveness and the capability of combining different types of measurement within FEMU, without having to decide which one to put more weight to. Furthermore, the EDMF revealed the engineering-acceptable candidate model sets and narrowed the updated variable ranges in the FE model. This suggests that relying solely on modal parameters (frequencies and/or mode shapes) is not recommended, particularly when the FE model will serve to simulate the response under traffic loads, for example, to support bridge structural safety analyses.

The future aim is to extend the proposed FEMU approach with B-WIM results. This will involve different magnitudes of traffic loading, even the extreme ones caused by the exceptional heavy vehicles; the recorded strain responses under the crossing heavy vehicles of known axle loads and configurations; the measured modal parameters; and the measured, not theoretical, influence lines. Finally, the strain and vibration measurements can be integrated into long-term monitoring systems, providing simultaneous strains and modal parameters to allow for more reliable identification and variation of the mode shapes.

## Figures and Tables

**Table 1:** Axle loads, axle spacing, and gross vehicle weights (GVWs) of the calibration vehicles.

	1st Axle		2nd Axle		3rd Axle		4th Axle		5th Axle	
Vehicle	Load [kN]	Spacing [m]	Load [kN]	Spacing [m]	Load [kN]	Spacing [m]	Load [kN]	Spacing [m]	Load [kN]	GVW [kN]
V1	67.69	3.30	85.35	1.35	88.29	/	/	/	/	241.33
V2	68.67	3.60	93.20	5.60	76.52	1.30	75.54	1.30	76.52	390.44
V3	68.67	3.30	87.31	1.35	87.31	5.17	76.52	1.33	76.52	396.32

**Table 2:** The number of signals (*n*), means, standard deviations (STDs), and coefficients of variation (CVs) for maximum measured values of calibration vehicle passages in lane L1.

*n*, Mean [μm/m], STD [μm/m], CV [%]		V1	V2	V3
SG_01	*n*	32	34	36
	Mean	19.1	29.5	31.5
	STD	0.7	0.9	1.3
	CV	3.5	2.9	4.2
SG_02	*n*	48	51	54
	Mean	27.1	35.4	37.9
	STD	1.3	1.2	1.4
	CV	4.8	3.4	3.7
SG_03	*n*	48	51	54
	Mean	27.9	35.5	36.8
	STD	1.5	1.3	1.6
	CV	5.3	3.5	4.4
SG_04	*n*	48	51	54
	Mean	18.2	27.2	27.4
	STD	0.9	1.2	1.6
	CV	5.2	4.6	5.7

**Table 3:** All identified natural frequencies and corresponding mode shapes from the experimental campaign. Red color indicates the greatest magnitude of displacements, while blue indicates the lowest.

T-1	B-1	B-2	MG_B-1	B-3
3.22 Hz	3.32 Hz	10.65 Hz	13.67 Hz	20.31 Hz
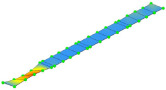	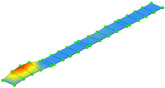	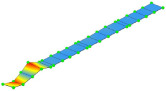	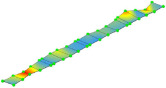	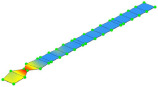
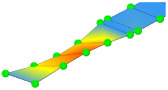	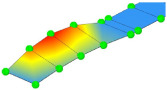	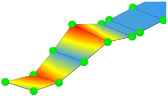	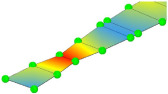	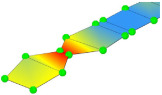

**Table 4:** Material properties of structural elements according to design documentation [33,42].

Element	Abbreviation	Young’s Modulus [GPa]	Poisson Ratio	Density [t/m^3^] ^1^
Piers	/	34	0.20	2.500
Slab	SLAB	33	0.20	2.500
External main girders	EMG (MG1, MG4)	35	0.20	2.575
Internal main girders	IMG (MG2, MG3)	34	0.20	2.575
Cross-girders	CG	35	0.20	2.500
Safety barriers 1	SB1	33	0.20	2.500
Safety barriers 2	SB2	33	0.20	2.500
Edge beams	EB	33	0.20	2.500
Asphalt	ASPH	8	0.35	2.582

^1^ Density of the main girders is increased due to the large number of prestressing tendons and mass of the equipment/installation attached to the main girders.

**Table 5:** Mesh convergence study of the natural frequencies for the M1_FULL_INIT FE model.

Mode	Natural Frequencies [Hz] for 0.25 m Global Element Size	Natural Frequencies [Hz] for 0.50 m Global Element Size
B-1	3.00	3.00
B-2	9.81	9.80
MG_B-1	12.83	12.78
B-3	20.39	20.37

**Table 6:** Comparison of natural frequencies and corresponding mode shapes of the M1_SUBSTR_INIT FE model and experimental values. Red color indicates the greatest magnitude of displacements, while blue indicates the lowest.

	Mode	B-1	B-2	MG_B-1	B-3
FE model M1_SUBSTR_INIT	Frequency	3.00 Hz	9.83 Hz	13.07 Hz	20.48 Hz
	Mode Shape (Full)	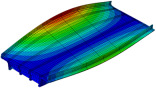	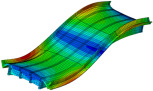	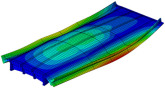	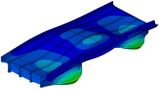
	Mode Shape (Z Cut)	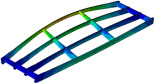	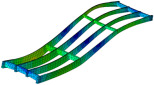	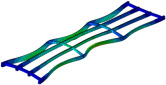	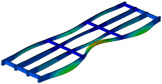
Experiment	Frequency	3.32 Hz	10.65 Hz	13.67 Hz	20.31 Hz
	Mode Shape	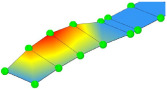	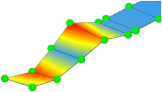	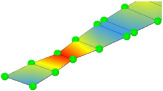	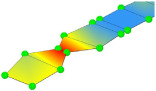

**Table 7:** Maximum strains in the M1_SUBSTR_INIT FE model compared to the mean and STD (standard deviation) values of maximum measured strains in sensors SG_01, SG_02, SG_03, and SG_04 under calibration vehicles V1, V2, and V3.

Strains [μm/m]		V1		V2		V3	
		Mean	STD	Mean	STD	Mean	STD
SG_01	M1_SUSBSTR_INIT	21.0	/	31.4	/	33.4	/
	Experiment	19.1	0.7	29.5	0.9	31.5	1.3
SG_02	M1_SUSBSTR_INIT	34.1	/	44.7	/	46.6	/
	Experiment	27.1	1.3	35.4	1.2	37.9	1.4
SG_03	M1_SUSBSTR_INIT	35.2	/	45.5	/	47.0	/
	Experiment	27.9	1.5	35.5	1.3	36.8	1.6
SG_04	M1_SUSBSTR_INIT	22.4	/	32.7	/	34.2	/
	Experiment	18.2	0.9	27.2	1.2	27.4	1.6

**Table 8:** List of variables considered in the sensitivity analysis with the description of modified variables.

Element/Variable/Property	Lower Value ^1^	Upper Value ^1^	Description
ASPH, SB1, SB2, EB, EMG (MG1, MG4), IMG (MG2, MG3), SLAB, CG	0.75 × design	1.25 × design	Young’s modulus change
BEARINGS TRANSL. STIFF.	0.75 × design	1.25 × design	Horizontal (X and Y) stiffness change
BEARINGS VERT. STIFF.	0.75 × design	1.25 × design	Vertical (Z) stiffness change
BEARINGS ROT. STIFF.	0.75 × design	1.25 × design	Rot. (around Y) stiffness change
DENSITY	0.95 × design	1.05 × design	Change in the density of elements ASPH, SB1, SB2, EB, EMG (MG1, MG4), IMG (MG2, MG3), SLAB, and CG

^1^ Design values from [33,42].

**Table 9:** Description of variables selected for FEMU and their range.

Variable	Description	Range	
		Res. Min.	EDMF
$\alpha_{ALL}$	Young’s modulus adjustment factor for ASPH, SB2, SB1, EB, EMG, IMG, SLAB, and CG	[0.9, 1.5]	/
$\alpha_{MG}$	Young’s modulus adjustment factor for EMG and IMG	[0.9, 1.5]	/
$\alpha_{OTHER}$	Young’s modulus adjustment factor for ASPH, SB2, SB1, EB, SLAB, and CG	[0.9, 1.5]	[0.10, 1.90]
$\alpha_{EMG}$	Young’s modulus adjustment factor for EMG	[0.9, 1.5]	[0.10, 2.00]
$\alpha_{IMG}$	Young’s modulus adjustment factor for IMG	[0.9, 1.5]	[0.10, 2.00]

**Table 10:** List of FEMU analyses describing variables, mode shapes/vehicles considered, and type of objective functions used.

Analysis Number	Analysis Type	Mode Shapes/Vehicles Considered	Variables	Acceleration-Based			$\mathrm{Strain}-\mathrm{Based}\;(J_{\varepsilon }$)
				$\mathrm{Frequency}-\mathrm{Based}\;(J_{f}$)	$\mathrm{MAC}-\mathrm{Based}\;(J_{MAC}$)	$\mathrm{Frequency}-\mathrm{and}-\mathrm{MAC}-\mathrm{Based}\;(J_{f,MAC}$)	
1	Res. min.	B-1, B-2, MG_B-1, B-3	$\alpha_{ALL}$	X			
2	Res. min.	B-1, B-2, MG_B-1, B-3	$\alpha_{ALL}$		X		
3	Res. min.	B-1, B-2, MG_B-1, B-3	$\alpha_{ALL}$			X	
4	Res. min.	V1, V2, V3	$\alpha_{ALL}$				X
5	Res. min.	B-1, B-2, MG_B-1, B-3	$\alpha_{MG}$ $,\;\alpha_{OTHER}$	X			
6	Res. min.	B-1, B-2, MG_B-1, B-3	$\alpha_{MG}$ $,\;\alpha_{OTHER}$		X		
7	Res. min.	B-1, B-2, MG_B-1, B-3	$\alpha_{MG}$ $,\;\alpha_{OTHER}$			X	
8	Res. min.	V1, V2, V3	$\alpha_{MG}$ $,\;\alpha_{OTHER}$				X
9	Res. min.	B-1, B-2, MG_B-1, B-3	$\alpha_{EMG}$ $,\;\alpha_{IMG}$ $,\;\alpha_{OTHER}$	X			
10	Res. min.	B-1, B-2, MG_B-1, B-3	$\alpha_{EMG}$ $,\;\alpha_{IMG}$ $,\;\alpha_{OTHER}$		X		
11	Res. min.	B-1, B-2, MG_B-1, B-3	$\alpha_{EMG}$ $,\;\alpha_{IMG}$ $,\;\alpha_{OTHER}$			X	
12	Res. min.	V1, V2, V3	$\alpha_{EMG}$ $,\;\alpha_{IMG}$ $,\;\alpha_{OTHER}$				X
13	EDMF	B-1, B-2, MG_B-1, B-3	$\alpha_{EMG}$ $,\;\alpha_{IMG}$ $,\;\alpha_{OTHER}$			X	
14	EDMF	V1, V2, V3	$\alpha_{EMG}$ $,\;\alpha_{IMG}$ $,\;\alpha_{OTHER}$				X
15	EDMF	B-1, B-2, MG_B-1, B-3 & V1, V2, V3	$\alpha_{EMG}$ $,\;\alpha_{IMG}$ $,\;\alpha_{OTHER}$			X	X

**Table 11:** Summary of FEMU results: values of variables that correspond to the best match within the last population.

Analysis Number	Variables	Acceleration-Based			Strain-Based
		Frequency-Based	MAC-Based	Frequency-and-MAC-Based	
1, 2, 3, 4	$\alpha_{ALL}$	1.18	0.99	1.18	1.21
5, 6, 7, 8	$\alpha_{MG}$ $,\;\alpha_{OTHER}$	1.20, 1.09	0.96, 0.90	0.97, 1.33	1.17, 1.50
9, 10, 11, 12	$\alpha_{EMG}$ $,\;\alpha_{IMG}$ $,\;\alpha_{OTHER}$	0.91, 1.50 1.17	0.90, 0.96, 1.02	1.36, 0.93, 1.09	0.90, 1.50, 1.01

**Table 12:** Results for all acceleration-based FEMU analyses: Values of measured, initial model’s (M1_SUBSTR_INIT), and updated model’s (M1_SUBSTR_UPDATE_*i*) natural frequencies.

Analysis Number (*i*)	Experiment [Hz]				M1_SUBSTR_INIT [Hz]				M1_SUBSTR_UPDATE_*i* [Hz]			
	B-1	B-2	MG_B-1	B-3	B-1	B-2	MG_B-1	B-3	B-1	B-2	MG_B-1	B-3
1	3.32	10.65	13.67	20.31	3.00	9.83	13.07	20.48	3.20	10.35	14.06	21.33
2									2.95	9.69	13.05	20.22
3									3.19	10.35	14.06	21.33
5									3.25	10.36	13.59	21.43
6									2.93	9.59	12.39	20.13
7									3.08	10.18	14.14	20.80
9									3.24	10.31	13.59	20.86
10									2.94	9.67	13.06	20.14
11									3.17	10.33	13.59	21.04

**Table 13:** Initial ranges of variables.

Variable	Initial Range
$\alpha_{EMG}$	[0.10, 0.20, …, 0.90, 0.95, …, 1.50, 1.60, …, 2.00]
$\alpha_{IMG}$	[0.10, 0.20, …, 0.90, 0.95, …, 1.50, 1.60, …, 2.00]
$\alpha_{OTHER}$	[0.10, 0.30, …, 0.50, 0.60, …, 1.30, 1.50, …, 1.90]

**Table 14:** Falsification thresholds for EDMF.

Analysis Number	Source		Falsification Thresholds ^1^	
			Min	Max
13	Acc.-Based	Frequencies	−5%, −5%, −5%, −5%	+5%, +5%, +5%, +5%
		MAC values	0.35, 0.20, 0.20, 0.20	0, 0, 0, 0
14	Strain-Based	Strains	−10%, −10%, −10%, −10%	+10%, +10%, +10%, +10%
15	Acc.-and-Strain-Based	Frequencies	−5%, −5%, −5%, −5%	+5%, +5%, +5%, +5%
		MAC values	0.35, 0.20, 0.20, 0.20	0, 0, 0, 0
		Strains	−10%, −10%, −10%, −10%	+10%, +10%, +10%, +10%

^1^ For frequencies, the threshold is defined as deviation in percentage from the measured frequencies; for MAC values, it is defined as absolute deviation from 1.0; and for strains, it is defined as deviation in percentage from the measured strains.

**Table 15:** Variable ranges: initial and after EDMF.

Variable	Initial Range	Range after EDMF		
		Analysis No. 13	Analysis No. 14	Analysis No. 15
		Acceleration-Based	Strain-Based	Acceleration-and-Strain-Based
	n = 9464	n = 35	n = 199	n = 7
$\alpha_{EMG}$	[0.10, 2.00]	[0.90, 1.60]	[0.40, 1.20]	[0.90, 1.10]
$\alpha_{IMG}$	[0.10, 2.00]	[0.80, 1.60]	[1.05, 2.00]	[1.25, 1.50]
$\alpha_{OTHER}$	[0.10, 1.90]	[0.90, 1.10]	[0.30, 1.90]	[1.00, 1.10]

## Data Availability

The finite element models and the data from the case study are available upon request from the corresponding author.
